# Construction and evaluation of a novel humanized HER2-specific chimeric receptor

**DOI:** 10.1186/bcr3674

**Published:** 2014-06-11

**Authors:** Meili Sun, Huan Shi, Chuanyong Liu, Jie Liu, Xianqiang Liu, Yuping Sun

**Affiliations:** 1 Department of Oncology, Jinan Central Hospital Affiliated to Shandong University, No.105, Jiefang Road, Jinan, Shandong 250013, People’s Republic of China; 2 Department of Oncology, Shandong Cancer Hospital and Institute, No.440 Jiyan Road, Jinan, Shandong 250117, People’s Republic of China; 3 Department of Breast and Thyroid Surgery, Jinan Central Hospital Affiliated to Shandong University, No.105, Jiefang Road, Jinan, Shandong 250013, People’s Republic of China

## Abstract

**Introduction:**

The human epidermal growth factor receptor 2 (HER2) represents one of the most studied tumor-associated antigens (TAAs) for cancer immunotherapy. The monoclonal antibody (mAb) trastuzumab has improved the outcomes of patients with HER2+ breast cancer. However, a large number of HER2+ tumors are not responsive to, or become resistant to, trastuzumab-based therapy, and thus more effective therapies targeting HER2 are needed.

**Methods:**

HER2-specific T cells were generated by the transfer of genes that encode chimeric antigen receptor (CAR). Using a multistep overlap extension PCR method, we constructed a novel, humanized HER2 CAR-containing, chA21 single-chain variable fragment (scFv) region of antigen-specific mAb and T-cell intracellular signaling chains made up of CD28 and CD3ζ. An interferon γ and interleukin 2 enzyme-linked immunosorbent assay and a chromium-51 release assay were used to evaluate the antitumor immune response of CAR T cells in coculture with tumor cells. Furthermore, SKBR3 tumor–bearing nonobese diabetic/severe combined immunodeficiency (NOD/SCID) mice were treated with HER2 CAR T cells to evaluate antitumor activity. Human CD3+ T cell accumulation in tumor xenograft was detected by immunohistochemistry.

**Results:**

chA21-28z CAR was successfully constructed, and both CD4+ and CD8+ T cells were transduced. The expanded HER2 CAR T cells expressed a central memory phenotype and specifically reacted against HER2+ tumor cell lines. Furthermore, the SKBR3 tumor xenograft model revealed that HER2 CAR T cells significantly inhibited tumor growth *in vivo*. Immunohistochemical analysis showed robust accumulation of human CD3+ T cells in regressing SKBR3 lesions.

**Conclusions:**

The results of this study show that novel chA21 scFv-based, HER2-specific CAR T cells not only recognized and killed HER2+ breast and ovarian cancer cells *ex vivo* but also induced regression of experimental breast cancer *in vivo*. Our data support further exploration of the HER2 CAR T-cell therapy for HER2-expressing cancers.

## Introduction

Human epidermal growth factor receptor 2 (HER2; also called Her-2/neu or ErbB2) is a member of the transmembrane epidermal growth factor receptor family and is one of the most studied tumor-associated antigens (TAAs) for cancer immunotherapy [1]. The monoclonal antibody (mAb) trastuzumab has improved the outcomes of patients with breast cancer who overexpress HER2 [2]. However, a consistent number of HER2+ tumors are not responsive to, or become resistant to, trastuzumab-based therapy, suggesting the need to develop additional novel therapies targeting HER2 [3]. Adoptive transfer of autologous tumor-reactive, tumor-infiltrating lymphocytes (TILs) can cause regression of solid tumors [4,5]. However, a major obstacle in the more widespread application of TIL therapy is the difficulty in identifying antigen-specific T cells in most human tumors [6]. Methods have been developed to engineer peripheral blood T cells to express a chimeric antigen receptor (CAR) [7,8]. CARs can be constructed by attaching a single-chain variable fragment (scFv) region of antigen-specific mAb to T-cell intracellular signaling chains. Transducing the resultant molecule into T cells enables these effector cells to recognize targets using antigen recognition of the antibody, and CARs are therefore major histocompatibility complex (MHC)–independent. The CAR approach combines the antigen specificity of an antibody with the ability to kill tumor cells mediated by T cells in a single fusion molecule [7-9]. Furthermore, CAR-modified T cells actively and specifically home to tumor sites and possess the capacity to expand and persist over the long term after tumor recognition *in vivo*. As such, CAR-modified T cells targeted to TAAs may be more effective than mAbs in generating durable tumor responses. In addition, it has been demonstrated that new-generation CARs containing the costimulatory signal domain of CD28 and/or 4-1BB, or other costimulatory molecules, enhance the function of the gene-modified T cells [10,11].

To date, the majority of CARs studied in clinical trials have been reconstructed with murine scFv specific for the TAAs, which have been shown to trigger a host immune response and thereby accelerate T-cell clearance, which has limited their success [12,13]. Hence, CARs containing both optimized costimulatory signaling domains and humanized or fully human scFv may be best suited for clinical trials of CAR-based T-cell therapy for cancer patients.

In the present study, we constructed a novel humanized anti-HER2 chA21 scFv-based CAR containing a fused CD28/CD3ζ endodomain (designated chA21-28z CAR) to achieve full T-cell activation. Our results show that the chA21-28z CAR T cells specifically reacted against HER2+ tumor cells *in vitro* and that these T cells caused dramatic inhibition of established HER2+ tumor cells after systemic administration of genetically redirected human T cells *in vivo*.

## Methods

### Cells

The human breast cancer cell lines SKBR3, MCF-7, T47D and MDA-MB-231 and the ovarian cancer cell lines SKOV3, OVCAR3, A2780 and A1847 were purchased from the American Type Culture Collection (ATCC; Manassas, VA, USA). TC-1, a human papillomavirus type 16–transformed mouse lung epithelial cell line obtained from ATCC, was used as a negative control for human HER2 expression. All tumor cell lines were cultured in complete medium composed of RPMI 1640, 10% heat-inactivated fetal bovine serum, 100 U/ml penicillin, 100 μg/ml streptomycin and 2 mM glutamine (all from Invitrogen, Carlsbad, CA, USA). Peripheral blood mononuclear cells (PBMCs) were cultured in the same medium with the addition of 100 U/ml human recombinant interleukin 2 (IL-2) (PeproTech, Suzhou, China) at 37°C and 5% CO_2_.

### Generation of a chimeric antigen receptor construct

A PEE14-chA21 plasmid containing humanized anti-HER2 chA21 scFv, described previously [14,15], was kindly provided by Prof Jing Liu (School of Life Science, University of Science and Technology of China). The CAR construct was generated through gene splicing by multistep overlap extension PCR (OE-PCR). The primers used are summarized in Table 1. The chA21-28z CAR was constructed based on one described in a previously published paper [10,16]. It consisted of a human CD8a leader sequence (amino acids 1 to 21), chA21 scFv, the human CD8a hinge region (amino acids 138 to 182), CD28 transmembrane and cytoplasmic domains (amino acids 153 to 220) and the T-cell receptor CD3*ζ* chain (amino acids 52 to 164). Briefly, in the first round of conventional PCRs using the Platinum *Taq* DNA Polymerase High Fidelity kit (Invitrogen), CD8a hinge region, CD28 and CD3ζ were amplified using the primer pairs CF and CR, DF and DR, and EF and ER, respectively. The human CD8a leader peptide fragment was synthesized (Takara Bio, Otsu, Japan) and then fused to chA21 scFv using the primers AF and BR by OE-PCR. A fragment encoding the CD8a hinge region was fused to CD28 using primers CF and DR, and then the PCR product (CD8a hinge + CD28) was fused to CD3z using primers CF and ER. These two PCR products were combined, and the full-length construct was generated using the AF and ER primers. The DNA encoding the full-length construct was ligated into the pSin lentiviral backbone (Addgene, Cambridge, MA, USA) to create the pSin-chA21-28Z plasmid.

**Table 1:** Primer sequences for HER2 chimeric antigen receptor construct

**Gene names**	**Primer names**	**Primer sequences (5′-3′)**
CD8a leader	AF	gcg*gaattc*atggccttaccagtgaccgccttgctcctgccg (*Eco*RI)
Her2scFv	BF	tgctccacgccgccaggccgagatctgacattgtgctgacccaaac
	BR	tgacgagacggtgactgaggttcc
CD8a hinge	CF	cctcagtcaccgtctcgtcaaccacgacgccagcgccgcg
	CR	atcacaggcgaagtccagccccc
CD28TM + ICD	DF	gggggctggacttcgcctgtgatttttgggtgctggtggtggttg
	DR	ggagcgataggctgcgaagtcgc
CD3ζ	EF	gcgacttcgcagcctatcgctccagagtgaagttcagcaggagc
	ER	gcg*gtcgac*ttagcgagggggcagggcctg (SaI1)

*Note*: *Eco*R1 and Sal1 restriction enzyme sites are italicized.

### Lentivirus production and transduction of peripheral blood mononuclear cells

Lentiviral particles were produced by transfecting 293 T cells with the lentiviral expression plasmid and the packaging plasmids. Briefly, 293 T cells were seeded into a 75-cm^2^ flask, and Lipofectamine 2000 (Invitrogen) was used as the transfection reagent at a ratio of 1 μg of DNA to 1.5 μl of Lipofectamine, according to the manufacturer’s instructions. The following amounts of DNA per 75-cm^2^ flask were used: 11 μg of chA21-28z transgene plasmid, 3.5 μg of vesicular stomatitis virus G glycoprotein envelope encoding pMD.G plasmid, 5 μg of packaging pMDLg/p plasmid and 2.5 μg of Rev-expressing plasmid. Two collections of viral supernatant were made 24 and 48 hours after transfection. After filtering the collections through a 0.45-μm filter (EMD Millipore, Billerica, MA, USA), a 0.75-ml aliquot of viral vector was frozen at −80°C for later use.

PBMCs were isolated by Ficoll density gradient centrifugation and activated with anti-CD3/CD28 beads (Invitrogen). Twenty-four hours after activation, PBMCs were transduced with lentiviral vectors at a multiplicity of infection of 5 and expanded for approximately 2 weeks.

### Fluorescence-activated cell sorting analysis

Anti-human CD45, CD3, CD4 and CD8 antibodies were purchased from BioLegend (San Diego, CA, USA). Cell surface expression of HER2 was detected by fluorescein isothiocyanate anti-HER2 antibody (clone 24D2; BioLegend). Matched isotype control antibodies were used in all analyses. HER2-specific CAR expression was detected by R-Phycoerythrin-AffiniPure F(ab′)_2_ antigen-binding fragment goat anti-mouse antibody (Jackson ImmunoResearch Laboratories, West Grove, PA, USA). Flow cytometric data were analyzed using FlowJo version 7.2.5 software (TreeStar, Ashland, OR, USA).

### Enzyme-linked immunosorbent assay

A cytokine release assay was performed by coculture of 10^5^ T cells with 10^5^ target cells per well in triplicate in 96-well flat-bottom plates in a 200-μl volume of complete medium. In addition, wells containing T cells alone were used as negative controls. The plates were incubated at 37°C. For antigen-specific assays, triplicate wells of Nunc MaxiSorp MicroWell plates (BioLegend) were coated with 5 μg/ml HER2-Fc chimeric protein (R&D Systems, Minneapolis, MN, USA) or CD19-Fc chimeric protein (SPEED BioSystems, Rockville, MD, USA) in 200 μl of phosphate-buffered saline (PBS) overnight at 4°C. After three washings with PBS, 10^5^ nontransduced (NT) or CAR-transduced T cells were then added, followed by incubation at 37°C. After about 24 hours, cell-free supernatants were assayed for the presence of interferon γ (IFN-γ) or IL-2 with an enzyme-linked immunosorbent assay kit (BioLegend).

### Chromium-51 release assay

The ability of the T cells to lyse tumor target cells was measured using a standard 4-hour chromium-51 (^51^Cr) release assay. Briefly, 1 × 10^6^ target cells were labeled with 0.1 mCi (3.7 MBq) ^51^Cr and mixed with decreasing numbers of effector cells at effector-to-target ratios of 30:1, 10:1, 3:1 and 1:1. After 4-hour incubation, harvested supernatants were counted using a WIZARD2 Automatic Gamma Counter (PerkinElmer, Waltham, MA, USA). Target cells incubated in complete medium alone or in 1% Triton X-100 were used to determine spontaneous and maximal ^51^Cr release, respectively. The mean percentage of specific lysis of triplicate wells was calculated according to the following formula: Specific lysis (%) = [(Specific release − Spontaneous release)/(Maximal release − Spontaneous release)] × 100.

### Xenograft model of breast cancer

The animal studies were approved by the Animal Ethics Committee of Shandong University. Nonobese diabetic/severe combined immunodeficient (NOD/SCID) mice were purchased from Beijing HFK Bioscience Co (Beijing, China). Eight- to twelve-week-old mice were bred and maintained under pathogen-free conditions in-house according to protocols approved by the Shandong University Institutional Animal Care and Use Committee. Female NOD/SCID mice were inoculated subcutaneously with 2 × 10^6^ SKBR3 cells on the flank on day 0. Sixty percent of mice developed solid tumors, and five tumor-bearing mice per group were randomized before treatment. After tumors became palpable, human PBMCs were activated and transduced as described above. After 2 weeks of expansion, when tumor burden was approximately 300 mm^3^, 2 × 10^7^ T cells (70% CAR+) or NT T cells were injected intravenously into the mice, and this was repeated 7 days later. Tumor dimensions were measured with calipers, and tumor volumes were calculated using the following formula: *V =* ½ × (length × width^2^), where length is the greatest longitudinal diameter and width is the greatest transverse diameter.

### Immunohistochemistry

The mice were killed by carbon dioxide (CO_2_) inhalation, and the tumors were removed and frozen in Tissue-Tek O.C.T. compound at −80°C. A standard streptavidin horseradish immunoperoxidase method was used for human CD3 staining as described previously [16]. Briefly, 7-μm cryosections were fixed in cold acetone for 5 minutes at 4°C and blocked with a peroxidase blocking system for 10 minutes (Dako, Carpinteria, CA, USA). Routine immunostaining was carried out with an initial 30-minute incubation with 10% normal goat serum at room temperature (RT), primary rabbit anti-human CD3 for T cells (RM-9107; Thermo Scientific, Waltham, MA, USA) at 1:100 dilution at RT for 45 minutes, secondary biotinylated goat anti-rabbit antibody at 1:200 dilution at RT for 30 minutes and streptavidin-biotinylated horseradish peroxidase complex reagent (Dako) at RT for 30 minutes, followed by washing three times for 5 minutes each after each incubation. Sections were then exposed to DAB + chromogen (Dako) for 5 minutes at RT, counterstained with hematoxylin, dehydrated, cleared and mounted.

### Statistical analysis

All data are reported as means ± SD. Data were analyzed using GraphPad Prism version 5.00 for Windows (GraphPad Software, San Diego, CA, USA). Statistical analysis was performed by analysis of variance for the tumor burden (tumor volume). Student’s *t*-test was used to evaluate differences in cytokine secretion and specific cytolysis. *P*-values <0.05 were considered significant.

## Results

### Construction of HER2-specific chimeric receptor

**Figure 1:**
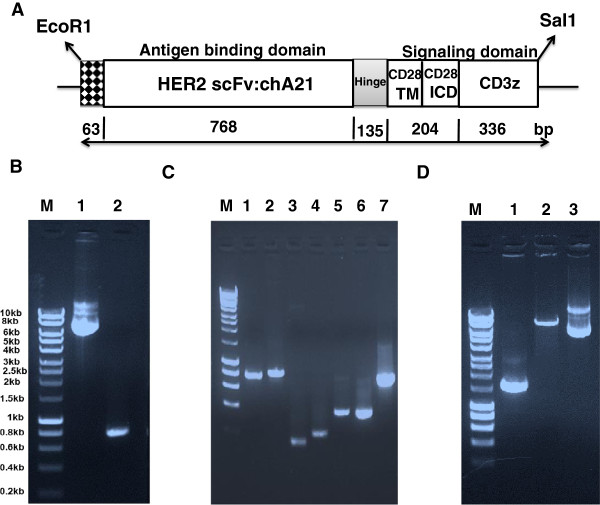
**Schematic diagram of the chA21-28z chimeric antigen receptor construct and chimeric gene products generated by overlap extension PCR. (A)** Diagram of the HER2-specific chimeric antigen receptor (CAR), consisting of a humanized chA21 single-chain variable fragment (scFv) linked to CD28 and CD3z signaling moieties. **(B)** Agarose gel electrophoresis of PCR products. Lane 1, PEE14-chA21 plasmid; lane 2, chA21 scFv. **(C)** Lane 1, A21 scFv; lane 2, CD8a leader + chA21 scFv; lane 3, CD8a hinge; lane 4, CD28TM + ICD; lane 5, CD8a hinge + CD28 (TM + ICD); lane 6, CD3z; lane 7, CD8a hinge + CD28 (TM + ICD) + CD3z. **(D)** Lane 1, chA21-28z CAR:CD8a leader + chA21 scFv + CD8a hinge + CD28 (TM + ICD) + CD3z; lane 2, pSin lentiviral vector; lane 3, pSin-chA21-28z CAR plasmid.

The humanized HER2-specific scFv chA21 was selected on the basis of its high binding affinity for HER2 (K_D_ = 11 nm) [17]. The affinity of chA21 scFv in the CAR construct is not known. HER2-specific CAR, consisting of chA21 scFv linked to CD28 and CD3z signaling moieties, was constructed and cloned into a lentiviral vector and named chA21-28z CAR (Figure 1A). The PCR products were constructed by fusion of independent fragments using an OE-PCR technique (Figures 1B, 1C and 1D). Briefly, in the first round of PCRs, the chA21 scFv (Figure 1B, lane 2) was amplified from PEE14-chA21 plasmid (Figure 1B, lane 1). The CD8a hinge region, CD28 and CD3ζ (Figure 1C, lanes 3, 4 and 6) were amplified from T-cell cDNA using conventional PCR conditions. Fragments generated by OE-PCR were also separated by electrophoresis to determine PCR product size. Figure 1C, lane 5: CD8a hinge CD28 (TM + ICD), lane 7: CD8a hinge CD28 (TM + ICD) CD3z; Figure 1D, lane 1: CD8a leader chA21 scFv CD8a hinge CD28 (TM + ICD) CD3z, lane 2: pSin lentiviral vector, lane 3: pSin-chA21-28z plasmid. The sequence of chA21-28z is given in Additional file 1: Figure S1, and the final plasmid was sequenced to confirm its identity.

### Expanded chimeric antigen receptor T cells express a central memory phenotype

**Figure 2:**
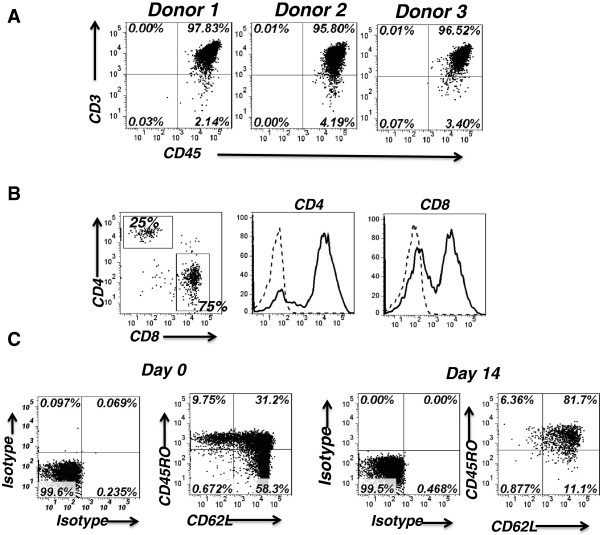
**The chA21-28z chimeric antigen receptor T cells cultured *in vitro *exhibit distinct populations defined by the differentiation markers CD45RO and CD62L. (A)** CD3+ T cells were the predominant cell population after 2 weeks of expansion. On day 14, peripheral blood mononuclear cells (PBMCs) cultured *in vitro* from three different donors contained more that 95% CD3 + CD45+ T cells. **(B)** HER2-specific chimeric antigen receptor (CAR) expression was detected by staining T cells with F(ab′)_2_ antigen-binding fragment anti-mouse antibody. Both CD4+ and CD8+ cells expressed HER2-specific CAR. **(C)** The T lymphocytes cultured *in vitro* were gated on CD3+ T cells and analyzed using the differentiation markers CD45RO and CD62L. The phenotypes of the cultured T cells from representative donors at days 0 and 14 are shown in dot plots.

Anti-CD3/CD28 bead-activated PBMCs from three healthy donors were transduced with HER2 CAR lentiviral vectors. After 14 days of culture, as shown in Additional file 2: Figure S2A and Figure 2A, more than 95% of the total cultured NT or CAR-transduced PBMCs were CD45 + CD3+ T cells, determined by flow cytometry, and most T cells expressed the CD8+ phenotype (Additional file 2: Figure S2B and Figure 2B). Activated CD8+ T cells generally proliferate faster than activated CD4+ T cells, and thus activation can promote a relative enrichment of CD8+ T cells in culture, which may explain the CD8+ phenotype of T cells. On average, approximately 70% of CD3+ T cells expressed HER2 CAR, which we determined by fluorescence-activated cell sorting (FACS) analysis. Both CD4+ and CD8+ T cells were transduced (Figure 2B). CD3+ NT and CAR T cells were further analyzed using the differentiation markers CD45RO and CD62L. Consistently, in the three donors tested, the central memory T (Tcm) cells were the least abundant at day 0, and the most plentiful at day 14, following transduction and *in vitro* culture (Additional file 2: Figure S2C and Figure 2C). Yang *et al*. [18] demonstrated that the phenotype of Tcm cells may be the most appropriate type for adoptive cell therapy.

### HER2 chimeric antigen receptor–modified T cells were specifically reactive to HER2+ tumor cell lines

**Figure 3:**
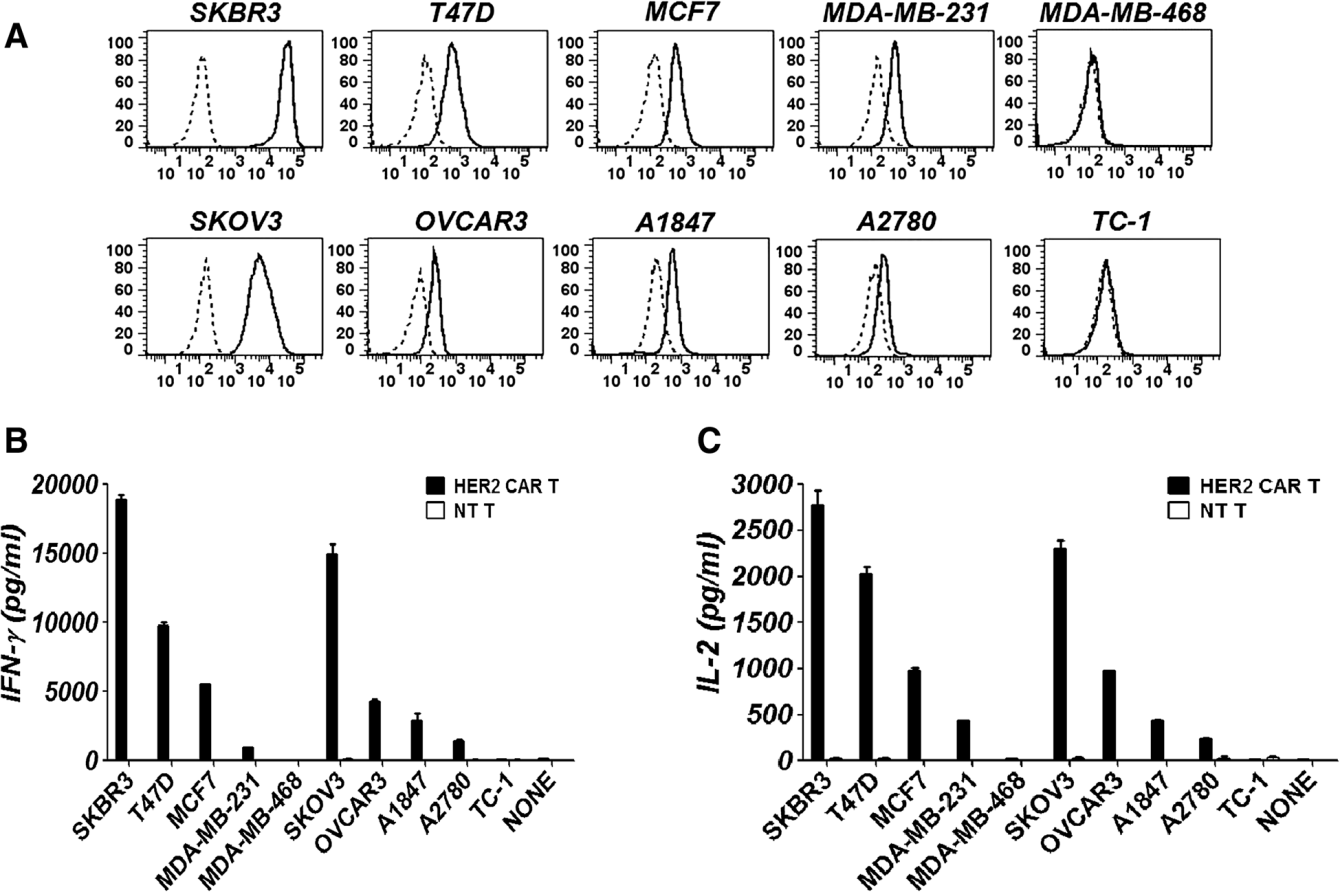
**Transduced chA21-28z chimeric antigen receptor T cells secrete interferon γ and interleukin 2 in response to tumor cells. (A)** Breast and ovarian cancer cell lines were stained with a monoclonal antibody specific for the HER2 antigen and analyzed by flow cytometry. HER2 expression was evident in the majority of cell lines after staining with an anti-HER2 monoclonal antibody (thick lines) compared to staining with control antibody (dashed lines). MDA-MB-468 and TC-1 were used as negative controls. **(B)** and **(C)** Antigen-specific interferon γ (IFN-γ) and interleukin 2 (IL-2) secretion by chA21-28z chimeric antigen receptor (CAR)–transduced T cells, but not nontransduced (NT) T cells, following overnight incubation with HER2+ cancer cell lines. Mean IFN-γ and IL-2 concentrations ± SEM (in pg/ml) from triplicate cultures are shown.

HER2 expression of a panel of tumor cell lines was examined by FACS and cell staining with an anti-HER2 antibody. The results show that HER2 expression was easily detected, not only for breast cancer cell lines SKBR3, MCF-7 and T47D but also for the ovarian cancer cell lines SKOV3, OVCAR3, A2780 and A1847. In addition, HER2 expression could be detected in a triple-negative breast cancer cell line, MDA-MB-231 (Figure 3A).

To test whether T cells expressing chA21-28z CAR were capable of specifically recognizing tumor lines expressing HER2, we coincubated CAR T cells with a panel of tumor cell lines and determined the amount of secreted cytokines IFN-γ and IL-2. chA21-28z CAR T cells recognized all of the HER2+ tumor lines and secreted IFN-γ and IL-2 at high levels. Very low levels of IFN-γ and IL-2 were observed when cocultured with the MDA-MB-468 and TC-1 cell lines, which we determined were negative for HER2 expression by FACS analysis. Only minimal IFN-γ and IL-2 production was detected when NT T cells were cocultured with HER2+ or HER2− cell lines (Figures 3B and 3C). To find out whether the recognition of HER2 by CAR T cells was antigen-specific, we tested whether coated HER2-Fc protein could activate CAR T cells. As shown in Additional file 3: Figure S3C, when incubated with HER2-Fc protein, but not CD19-Fc protein, HER2 CAR T cells were activated and released IFN-γ at a high level.

**Figure 4:**
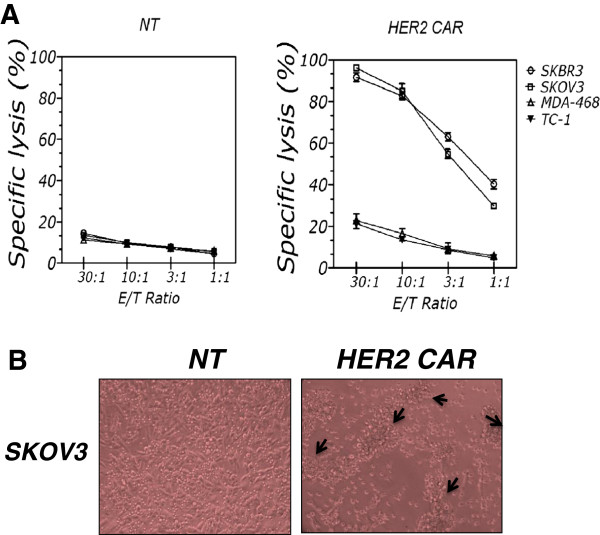
**Specific lysis by chA21-28z chimeric antigen receptor T cells *in vitro*. (A)** Antigen-specific killing of HER2+ tumor cells by chA21-28z chimeric antigen receptor (CAR) T cells was determined using a 4-hour chromium-51 release assay at the indicated effector-to-target (E/T) ratio. Nontransduced (NT) T cells and HER2− tumor cells served as controls. **(B)** NT or chA21-28z CAR T cells were seeded into cultures of SKOV3 cells expressing HER2. After about 24 hours in coculture, formation of T-cell clusters and elimination of the SKOV3 monolayer were visible.

Next, we evaluated the ability of CAR T cells to mediate lysis of HER2+ tumor cells in a 4-hour ^51^Cr release assay. CAR T cells lysed HER2+ SKOV3, SKBR3 and T47D cells (Figure 4A), but not HER2− MDA-MB468 and TC-1 cells. Lysis of these tumor cells was dependent on expression of CAR, as no lysis by NT T cells was observed. In addition, we seeded NT or chA21-28z CAR T cells into cultures of SKOV3 cells expressing HER2. After 24 hours of T-cell seeding, formation of T-cell clusters and elimination of the SKOV3 monolayer were visible (Figure 4B). These results indicate that HER2-specific T cells recognize and kill HER2+ cells in a HER2-specific manner.

### Antitumor responses by HER2 chimeric antigen receptor T cells *in vivo*

**Figure 5:**
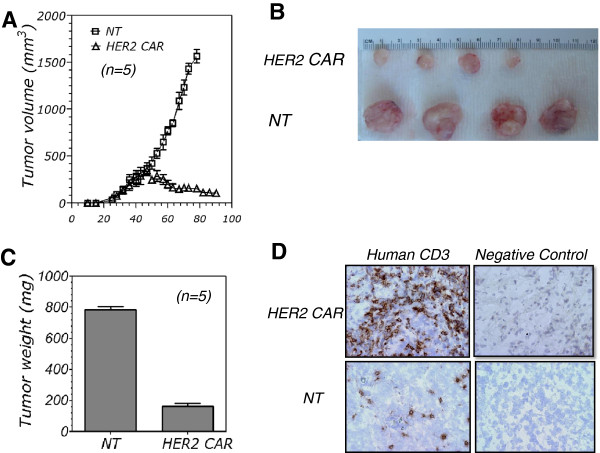
**chA21-28z chimeric antigen receptor T cells inhibit tumor growth *in vivo*. (A)** Two million SKBR3 tumor cells were injected subcutaneously into nonobese diabetic/severe combined immunodeficiency (NOD/SCID) mice, followed by intravenous treatment with T cells beginning on either day 40 or day 47 after tumor inoculation. Tumors regressed in response to injections of chA21-28z chimeric antigen receptor (CAR) T cells (triangles). Tumors grew progressively in mice (squares) that received nontransduced (NT) T-cell injections. HER2 CAR T cells inhibited tumor growth to a significantly greater extent compared to the control group (*P* = 0.00012). **(B)** SKBR tumors were dissected, and photographs of four representative samples are shown. **(C)** Graphed tumor weight data. Values are expressed as mean ± SEM. **(D)** SKBR3 tumors were collected from killed mice and stained for human CD3 expression (brown). Representative sections are shown at 100× original magnification.

Having determined the antitumor activity of chA21-28z CAR T cells *in vitro*, we next sought to discover their antitumor ability in tumor-bearing mice. We compared the potency of CAR T cells to that of NT T cells in terms of tumor volume in a xenograft model. A total of 2 × 10^6^ SKBR3 cells were subcutaneously injected into NOD/SCID mice, and tumor volume reached 300 mm^3^ after about 7 weeks. Treatment was performed weekly for 2 weeks, and tumor growth was monitored. chA21-28z CAR T cells elicited stronger antitumor responses than the responses in the control mice treated with NT T cells (*P* = 0.00012) (Figures 5A and 5B). The average tumor weight of the control group was 0.79 ± 0.03 g, and that of the CAR T cell–treated group was 0.16 ± 0.02 g (*P* = 0.0023) (Figure 5C) at day 92 after inoculation of tumor cells, indicating that CAR T cells significantly inhibited tumor growth.The infiltration of tumor-reactive T cells into tumors was highly correlated with tumor regression. Immunohistochemical analysis revealed robust accumulation of human CD3+ T cells in regressing SKBR3 lesions 7 weeks after intravenous T-cell injection, although few CD3+ T cells were detected in tumors that received NT T-cell injections (Figure 5D).

## Discussion

Our goal in this study was to construct and evaluate a novel anti-HER2 chA21 scFv-based CAR. CARs are recombinant receptors that couple antigen-specific targeting of an extracellular antibody, scFv, with intracellular T-cell signaling domains for the generation of antigen-redirected T cells for therapy. We utilized an OE-PCR technique to generate a linearized, HER2-specific CAR that contains a CD28 costimulatory signaling domain. OE-PCR provides a rapid, cost-effective means of creating chimeric receptors without the need for available enzyme recognition sequences [19,20]. Furthermore, PCR-based methods have become increasingly reliable with the introduction of high-fidelity DNA polymerases, thus limiting the number of unwanted mutations in the final construct [19-21]. This method has proven to be useful for the construction of CARs containing various costimulatory signaling domains, and these CARs can simply be cloned into a standard plasmid for bacterial propagation.

Various costimulatory domains have been tested in CAR constructs, including CD28, 41BB, CD27, ICOS and OX40 [11,22-24]. Notably, CD28 costimulation augments antiapoptotic Bcl**-**X_L_ expression [25], increases IL-2-independent proliferation, enhances the resistance of CAR T cells to T regulatory cells [26] and is the costimulatory domain choice for most CARs. In clinical studies, antitumor activity and the persistence of CAR T cells can be enhanced by adding a CD28 costimulatory domain [27]. Therefore, in our study, we added the signaling domain of CD28 to the cytoplasmic domain of the chA21 scFv CAR domain to achieve full T-cell activation.

chA21 scFv was derived from chA21 mAb, which may represent another group of anti-ErbB2 antibody. chA21 mAb recognizes epitopes that are located mainly in subdomain I and apart from the functional sites for HER2 receptor dimerization, whereas trastuzumab (4D5) binds to the juxtamembrane region in subdomain IV [28]. Importantly, researchers in previous studies [29-31] have demonstrated that the chA21 mAb can inhibit growth and induce apoptosis of the human ovarian cancer cell line SKOV3 by regulating the balance between Bax and Bcl-2, suggesting that chA21 mAb might be a promising new candidate in the treatment of HER2-overexpressing cancers.

In this study, we found that human T cells expressing chA21 scFv-28z-based CARs lysed HER2+ breast and ovarian cancer cells with high efficiency. CAR T cells recognized and killed tumor cells in an MHC-independent manner, so that target cell recognition by CAR T cells was unaffected by downregulation of human leukocyte antigen class I molecules and defective antigen processing. Besides tumor cell killing, cytokines production of CAR T cells suggest their activation and sustained antitumor activity. chA21-28z CAR T cells were capable of producing greater amounts of IFN-γ and IL-2 compared to NT T cells. IFN-γ produced by cytotoxic T cells is critical for exerting immune surveillance of tumors, which can directly inhibit proliferation and induce apoptosis of some malignancies *in vivo* and *in vitro* through elusive mechanisms [32]. IL-2 is pivotal for the proliferation and differentiation of T cells to become effector T cells. Moreover, two systemic injections of chA21-28z T cells against subcutaneous tumors led to dramatic tumor regression in mice. This type of model is generally accepted to be a test of therapy more stringent than other models that involve injection of T cells directly into tumors. T-cell infiltration into tumors was attainable by systemic T-cell delivery, showing the capacity of transferred T cells to circulate, home to the tumor and perform antitumor functions. In addition, humanized chA21 scFv-based CAR T cells may greatly reduce their immunogenic potential *in vivo*. However, even fully human antibodies may lead to the production of human antihuman antibodies, and only use in a clinical trial will prove conclusively whether they are nonimmunogenic.

HER2 CAR T-cell therapies have been tested in various cancer models in mice, including breast cancer [33], ovarian cancer [34], medulloblastoma [35], glioblastoma [36] and osteosarcoma [37]. Zhao *et al*. [33] reported that trastuzumab-based 4D5 CAR T cells reacted against a panel of tumor cells of different origin that expressed even low levels of HER2, as well as multiple normal cells. In a clinical setting, transfer of high numbers of 4D5 CAR T cells encompassing CD28 and CD137 costimulatory domains following lymphodepletion was reported to result in severe toxicity, including cytokine storm, respiratory distress and ultimately patient death due to the CAR T cells’ response against lung epithelial cells expressing low levels of HER2 [38]. In the future, we will compare the antitumor activity of adoptive HER2 CAR T-cell therapy with passive immunotherapy with trastuzumab. Importantly, incorporating a suicide gene, such as the inducible caspase 9 suicide gene system, within engineered CAR T cells would provide a safety control for clinical application [39]. Moreover, injections of transient CAR-expressing T cells electroporated with mRNAs appear to be safe, and the toxicity would be expected to abate rapidly [40].

## Conclusions

Collectively, we constructed an HER2-specific CARs using a novel, humanized HER2 scFv. We demonstrate for the first time, to the best of our knowledge, that a novel chA21 scFv-based, HER2-specific CAR T cell not only recognized and killed HER2+ breast and ovarian cancer cells *ex vivo* but also induced regression of experimental breast cancer *in vivo*. Hence, the adoptive transfer of HER2-redirected T cells may be a viable immunotherapeutic approach for treating HER2-expressing cancers.

## Abbreviations

CAR: Chimeric antigen receptor; ELISA: Enzyme-linked immunosorbent assay; HER2: Human epidermal growth factor receptor 2; IHC: Immunohistochemistry; IFN: Interferon; IL: Interleukin; mAb: Monoclonal antibody; MHC: Major histocompatibility complex; OE-PCR: Overlap extension polymerase chain reaction; PBMC: Peripheral blood mononuclear cell; PBS: Phosphate-buffered saline; RT: Room temperature; ScFv: Single-chain variable fragment; TAA: Tumor-associated antigen; TIL: Tumor-infiltrating lymphocyte.

## Competing interests

The authors declare that they have no competing interests.

## Authors’ contributions

YS contributed to the study conception, experimental design, data analysis and manuscript writing. MS and HS carried out all the *in vitro* experiments and most of the *in vivo* experiments. CL and JL offered technical support in the animal studies. CL, JL and XL carried out IHC staining and reviewed the IHC slides. MS, HS and YS drafted the manuscript. MS and SY provided funding, supervised the research and finalized the manuscript. All authors read and approved the final manuscript.

## Supplementary Material

Additional file 1: Figure S1 Nucleotide and amino acid sequences of chA21-28z CAR construct. 1 to 6 bp: *Eco*RI; 7 to 69 bp: CD8a leader; 70 to 837 bp: chA21 scFv; 838 to 972 bp: CD8a hinge; 973 to 1,059 bp: CD28 transmembrane; 1,060 to 1,176 bp: CD28 intracellular domain; 1,177 to 1,512 bp: TCR-CD3z intracellular domain; 1,513 to 1,515 bp: Stop codon; 1,516 to 1,521: *Sal*I. Click here for file

Additional file 2: Figure S2 The phenotype of NT T cells cultured *in vitro*. **(A)** CD3+ T cells were the predominant cell population after 2 weeks of expansion. On day 14, PBMCs from three different donors cultured *in vitro* contained more that 95% CD3 + CD45+ T cells. **(B)** Most T cells expressed the CD8+ phenotype. **(C)** The NT T lymphocytes cultured *in vitro* were gated on CD3+ T cells and analyzed using differentiated markers CD45RO and CD62L. The phenotype of the cultured T cells from a representative donor at days 0 and 14 is shown in dot plots. Click here for file

Additional file 3: Figure S3 The recognition of HER2 by CAR T cells was antigen-specific. Triplicate wells of Nunc MaxiSorp MicroWell plates were coated with 5 μg/ml HER2-Fc chimeric protein or CD19-Fc chimeric protein in 200 μl of PBS overnight at 4°C. After three washings with PBS, 10^5^ NT or CAR T cells were added, followed by incubation at 37°C. After about 24 hours, cell-free supernatants were assayed for the presence of IFN-γ. Click here for file

## References

[B1] Arteaga CL Sliwkowski MX Osborne CK Perez EA Puglisi F Gianni L Treatment of HER2-positive breast cancer: current status and future perspectives Nat Rev Clin Oncol 2011 9 16 32 22124364 10.1038/nrclinonc.2011.177

[B2] Hortobagyi GN Trastuzumab in the treatment of breast cancer N Engl J Med 2005 353 1734 1736 16236745 10.1056/NEJMe058196

[B3] Valabrega G Montemurro F Aglietta M Trastuzumab: mechanism of action, resistance and future perspectives in HER2-overexpressing breast cancer Ann Oncol 2007 18 977 984 17229773 10.1093/annonc/mdl475

[B4] Rosenberg SA Yang JC Sherry RM Kammula US Hughes MS Phan GQ Citrin DE Restifo NP Robbins PF Wunderlich JR Durable complete responses in heavily pretreated patients with metastatic melanoma using T-cell transfer immunotherapy Clin Cancer Res 2011 17 4550 4557 21498393 10.1158/1078-0432.CCR-11-0116 PMC3131487

[B5] Dudley ME Wunderlich JR Yang JC Sherry RM Topalian SL Restifo NP Royal RE Kammula U White DE Mavroukakis SA Adoptive cell transfer therapy following non-myeloablative but lymphodepleting chemotherapy for the treatment of patients with refractory metastatic melanoma J Clin Oncol 2005 23 2346 2357 15800326 10.1200/JCO.2005.00.240 PMC1475951

[B6] Chinnasamy N Morgan RA Recent progress and future directions using engineered T cells for the treatment of cancer Gene Ther Regul 2010 5 67 80

[B7] Liu L Sun M Wang Z Adoptive T-cell therapy of B-cell malignancies: conventional and physiological chimeric antigen receptors Cancer Lett 2012 316 1 5 22099879 10.1016/j.canlet.2011.10.027

[B8] Gross G Eshhar Z Endowing T cells with antibody specificity using chimeric T cell receptors FASEB J 1992 6 3370 3378 1464371 10.1096/fasebj.6.15.1464371

[B9] Shi S Wang R Chen Y Song H Chen L Huang G Combining antiangiogenic therapy with adoptive cell immunotherapy exerts better antitumor effects in non-small cell lung cancer models PLoS One 2013 8 e65757 23799045 10.1371/journal.pone.0065757 PMC3683034

[B10] Carpenito C Milone MC Hassan R Simonet JC Lakhal M Suhoski MM Varela-Rohena A Haines KM Heitjan DF Albelda SM Control of large, established tumor xenografts with genetically retargeted human T cells containing CD28 and CD137 domains Proc Natl Acad Sci U S A 2009 106 3360 3365 19211796 10.1073/pnas.0813101106 PMC2651342

[B11] Song DG Ye Q Poussin M Harms GM Figini M Powell DJ CD27 costimulation augments the survival and antitumor activity of redirected human T cells in vivo Blood 2012 119 696 706 22117050 10.1182/blood-2011-03-344275

[B12] Kershaw MH Westwood JA Parker LL Wang G Eshhar Z Mavroukakis SA White DE Wunderlich JR Canevari S Rogers-Freezer L A phase I study on adoptive immunotherapy using gene-modified T cells for ovarian cancer Clin Cancer Res 2006 12 6106 6115 17062687 10.1158/1078-0432.CCR-06-1183 PMC2154351

[B13] Lamers CH Langeveld SC Groot-van Ruijven CM Debets R Sleijfer S Gratama JW Gene-modified T cells for adoptive immunotherapy of renal cell cancer maintain transgene-specific immune functions in vivo Cancer Immunol Immunother 2007 56 1875 1883 17479266 10.1007/s00262-007-0330-3 PMC11030170

[B14] Cheng LS Ai Ping L Jia Hong Y Yan Qiu D Liang Wei L Jing W Chao Chen W Jing L Construction, expression and characterization of the engineered antibody against tumor surface antigen, p185c-erbB-2 Cell Res 2003 13 35 48 12643348 10.1038/sj.cr.7290149

[B15] Wang J Shi Y Liu Y Hu S Ma J Liu J Cheng L Purification and characterization of a single-chain chimeric anti-p185 antibody expressed by CHO–GS system Protein Expr Purif 2005 41 68 76 15802223 10.1016/j.pep.2004.11.007

[B16] Song DG Ye Q Carpenito C Poussin M Wang LP Ji C Figini M June CH Coukos G Powell DJ *In vivo* persistence, tumor localization, and antitumor activity of CAR-engineered T cells is enhanced by costimulatory signaling through CD137 (4-1BB) Cancer Res 2011 71 4617 4627 21546571 10.1158/0008-5472.CAN-11-0422 PMC4140173

[B17] Hu S Li L Qiao J Guo Y Cheng L Liu J Codon optimization, expression, and characterization of an internalizing anti-ErbB2 single-chain antibody in *Pichia pastoris* Protein Expr Purif 2006 47 249 257 16403645 10.1016/j.pep.2005.11.014

[B18] Yang S Luca G Liu F Ji Y Yu Z Restifo NP Rosenberg SA Morgan RA In vitro generated anti-tumor T lymphocytes exhibit distinct subsets mimicking in vivo antigen-experienced cells Cancer Immunol Immunother 2011 60 739 749 21305379 10.1007/s00262-011-0977-7 PMC3080434

[B19] Heckman KL Pease LR Gene splicing and mutagenesis by PCR-driven overlap extension Nat Protoc 2007 2 924 932 17446874 10.1038/nprot.2007.132

[B20] Wurch T Lestienne F Pauwels PJ A modified overlap extension PCR method to create chimeric genes in the absence of restriction enzymes Biotechnol Tech 1998 12 653 657

[B21] Bryksin AV Matsumura I Overlap extension PCR cloning: a simple and reliable way to create recombinant plasmids Biotechniques 2010 48 463 20569222 10.2144/000113418 PMC3121328

[B22] Hombach AA Abken H Costimulation by chimeric antigen receptors revisited the T cell antitumor response benefits from combined CD28‒OX40 signalling Int J Cancer 2011 129 2935 2944 22030616 10.1002/ijc.25960

[B23] Finney HM Akbar AN Lawson AD Activation of resting human primary T cells with chimeric receptors: costimulation from CD28, inducible costimulator, CD134, and CD137 in series with signals from the TCRζ chain J Immunol 2004 172 104 113 14688315 10.4049/jimmunol.172.1.104

[B24] Shen CJ Yang YX Han EQ Cao N Wang YF Wang Y Zhao YY Zhao LM Cui J Gupta P Chimeric antigen receptor containing ICOS signaling domain mediates specific and efficient antitumor effect of T cells against EGFRvIII expressing glioma J Hematol Oncol 2013 6 33 23656794 10.1186/1756-8722-6-33 PMC3658918

[B25] Boise LH Minn AJ Noel PJ June CH Accavitti MA Lindsten T Thompson CB CD28 costimulation can promote T cell survival by enhancing the expression of Bcl-XL Immunity 1995 3 87 98 7621080 10.1016/1074-7613(95)90161-2

[B26] Loskog A Giandomenico V Rossig C Pule M Dotti G Brenner MK Addition of the CD28 signaling domain to chimeric T-cell receptors enhances chimeric T-cell resistance to T regulatory cells Leukemia 2006 20 1819 1828 16932339 10.1038/sj.leu.2404366

[B27] Savoldo B Ramos CA Liu E Mims MP Keating MJ Carrum G Kamble RT Bollard CM Gee AP Mei Z Liu H Grilley B Rooney CM Heslop HE Brenner MK Dotti G CD28 costimulation improves expansion and persistence of chimeric antigen receptor-modified T cells in lymphoma patients J Clin Invest 2011 121 1822 1826 21540550 10.1172/JCI46110 PMC3083795

[B28] Zhou H Zha Z Liu Y Zhang H Zhu J Hu S Shen G Cheng L Niu L Greene MI Structural insights into the down-regulation of overexpressed p185^*her2/neu*^ protein of transformed cells by the antibody chA21 J Biol Chem 2011 286 31676 31683 21680730 10.1074/jbc.M111.235184 PMC3173138

[B29] Yi G Qiang W Xiaoguang L Liansheng C Jing L Effect of anti-HER-2 engineered antibody chA21 on MMP-2 and TIMP-2 expression of human ovarian cancer SKOV3 cells J Xi’an Jiaotong Univ (Med Sci) 2007 4 006

[B30] Xue H Wu Q Hu X Ling X Yang F Cheng L Liu J Effects of anti-HER-2 chimeric antibody chA21 on proliferation and apoptosis of SKBR3 cells Chin Pharmacol Bull 2007 23 1463

[B31] Shen G Huang H Zhang A Zhao T Hu S Cheng L Liu J Xiao W Ling B Wu Q In vivo activity of novel anti-ErbB2 antibody chA21 alone and with paclitaxel or trastuzumab in breast and ovarian cancer xenograft models Cancer Immunol Immunother 2011 60 339 348 21086124 10.1007/s00262-010-0937-7 PMC11029528

[B32] Wall L Burke F Barton C Smyth J Balkwill F IFN-γ induces apoptosis in ovarian cancer cells *in vivo* and *in vitro* Clin Cancer Res 2003 9 2487 2496 12855622

[B33] Zhao Y Wang QJ Yang S Kochenderfer JN Zheng Z Zhong X Sadelain M Eshhar Z Rosenberg SA Morgan RA A Herceptin-based chimeric antigen receptor with modified signaling domains leads to enhanced survival of transduced T lymphocytes and antitumor activity J Immunol 2009 183 5563 5574 19843940 10.4049/jimmunol.0900447 PMC6292203

[B34] Yoon S Lee J Cho H Kim E Kim H Park M Kim T Adoptive immunotherapy using human peripheral blood lymphocytes transferred with RNA encoding Her-2/neu-specific chimeric immune receptor in ovarian cancer xenograft model Cancer Gene Ther 2008 16 489 497 19096447 10.1038/cgt.2008.98

[B35] Ahmed N Ratnayake M Savoldo B Perlaky L Dotti G Wels WS Bhattacharjee MB Gilbertson RJ Shine HD Weiss HL Regression of experimental medulloblastoma following transfer of HER2-specific T cells Cancer Res 2007 67 5957 5964 17575166 10.1158/0008-5472.CAN-06-4309

[B36] Ahmed N Salsman VS Kew Y Shaffer D Powell S Zhang YJ Grossman RG Heslop HE Gottschalk S HER2-specific T cells target primary glioblastoma stem cells and induce regression of autologous experimental tumors Clin Cancer Res 2010 16 474 485 20068073 10.1158/1078-0432.CCR-09-1322 PMC3682507

[B37] Rainusso N Brawley V Ghazi A Hicks M Gottschalk S Rosen J Ahmed N Immunotherapy targeting HER2 with genetically modified T cells eliminates tumor-initiating cells in osteosarcoma Cancer Gene Ther 2011 19 212 217 22173710 10.1038/cgt.2011.83

[B38] Morgan RA Yang JC Kitano M Dudley ME Laurencot CM Rosenberg SA Case report of a serious adverse event following the administration of T cells transduced with a chimeric antigen receptor recognizing ERBB2 Mol Ther 2010 18 843 851 20179677 10.1038/mt.2010.24 PMC2862534

[B39] Di Stasi A Tey SK Dotti G Fujita Y Kennedy-Nasser A Martinez C Straathof K Liu E Durett AG Grilley B Inducible apoptosis as a safety switch for adoptive cell therapy N Engl J Med 2011 365 1673 1683 22047558 10.1056/NEJMoa1106152 PMC3236370

[B40] Zhao Y Moon E Carpenito C Paulos CM Liu X Brennan AL Chew A Carroll RG Scholler J Levine BL Multiple injections of electroporated autologous T cells expressing a chimeric antigen receptor mediate regression of human disseminated tumor Cancer Res 2010 70 9053 9061 20926399 10.1158/0008-5472.CAN-10-2880 PMC2982929

