# Supplementary material for: Construction and evaluation of a novel humanized HER2-specific chimeric receptor
Source: Breast Cancer Res. 2014 Jun 11;16(3):R61. doi: 10.1186/bcr3674 (PMC4095682; doi:10.1186/bcr3674)
Supplement: Additional file 1: Figure S1 — Nucleotide and amino acid sequences of chA21-28z CAR construct. 1 to 6 bp: EcoRI; 7 to 69 bp: CD8a leader; 70 to 837 bp: chA21 scFv; 838 to 972 bp: CD8a hinge; 973 to 1,059 bp: CD28 transmembrane; 1,060 to 1,176 bp: CD28 intracellular domain; 1,177 to 1,512 bp: TCR-CD3z intracellular domain; 1,513 to 1,515 bp: Stop codon; 1,516 to 1,521: SalI. [file bcr3674-S1.docx]

Additional file 1: Figure S1

M A L P V T A L L L P L A L L L H A A R P G S D I V L T Q T P S ·

1 GAATTCATGG CCTTACCAGT GACCGCCTTG CTCCTGCCGC TGGCCTTGCT GCTCCACGCC GCCAGGCCGG GATCTGACAT TGTGCTGACC CAAACTCCAT

CTTAAGTACC GGAATGGTCA CTGGCGGAAC GAGGACGGCG ACCGGAACGA CGAGGTGCGG CGGTCCGGCC CTAGACTGTA ACACGACTGG GTTTGAGGTA

· S L P V S V G E K V T M T C K S S Q T L L Y S N N Q K N Y L A W Y ·

101 CCTCCCTACC TGTGTCAGTT GGAGAGAAGG TTACTATGAC CTGCAAGTCC AGTCAGACCC TTTTATATAG TAACAATCAA AAGAACTACT TGGCCTGGTA

GGAGGGATGG ACACAGTCAA CCTCTCTTCC AATGATACTG GACGTTCAGG TCAGTCTGGG AAAATATATC ATTGTTAGTT TTCTTGATGA ACCGGACCAT

· Q Q K P G Q S P K L L I S W A F T R K S G V P D R F T G S G S G T

201 CCAGCAGAAA CCAGGGCAGT CTCCTAAACT GCTGATTTCC TGGGCATTCA CTAGGAAATC TGGGGTCCCT GATCGCTTCA CAGGCAGTGG ATCTGGGACA

GGTCGTCTTT GGTCCCGTCA GAGGATTTGA CGACTAAAGG ACCCGTAAGT GATCCTTTAG ACCCCAGGGA CTAGCGAAGT GTCCGTCACC TAGACCCTGT

D F T L T I G S V K A E D L A V Y Y C Q Q Y S N Y P W T F G G G T R ·

301 GATTTCACTC TCACCATCGG CAGTGTGAAG GCTGAAGACC TGGCAGTTTA TTACTGTCAG CAATATTCTA ACTATCCGTG GACGTTCGGT GGAGGCACCA

CTAAAGTGAG AGTGGTAGCC GTCACACTTC CGACTTCTGG ACCGTCAAAT AATGACAGTC GTTATAAGAT TGATAGGCAC CTGCAAGCCA CCTCCGTGGT

· L E I K R G G G G S G G G G S G G G G S G G G G S E V Q L Q Q S G ·

401 GGCTGGAAAT CAAACGGGGT GGTGGTGGTT CTGGTGGTGG TGGTTCTGGC GGCGGCGGCT CCGGTGGTGG TGGATCCGAG GTCCAGCTGC AGCAGTCTGG

CCGACCTTTA GTTTGCCCCA CCACCACCAA GACCACCACC ACCAAGACCG CCGCCGCCGA GGCCACCACC ACCTAGGCTC CAGGTCGACG TCGTCAGACC

· P E V V K T G A S V K I S C K A S G Y S F T G Y F I N W V K K N S

501 ACCTGAGGTA GTGAAGACTG GGGCTTCAGT GAAGATATCC TGCAAGGCTT CTGGTTACTC ATTCACTGGT TACTTCATAA ACTGGGTCAA GAAGAACTCT

TGGACTCCAT CACTTCTGAC CCCGAAGTCA CTTCTATAGG ACGTTCCGAA GACCAATGAG TAAGTGACCA ATGAAGTATT TGACCCAGTT CTTCTTGAGA

G K S P E W I G H I S S S Y A T S T Y N Q K F K N K A A F T V D T S ·

601 GGAAAGAGCC CTGAGTGGAT TGGACACATT AGTTCTTCCT ATGCTACCTC TACCTACAAC CAGAAGTTTA AAAACAAGGC CGCATTTACT GTAGACACAT

CCTTTCTCGG GACTCACCTA ACCTGTGTAA TCAAGAAGGA TACGATGGAG ATGGATGTTG GTCTTCAAAT TTTTGTTCCG GCGTAAATGA CATCTGTGTA

· S S T A F M Q L N S L T S E D S A V Y Y C V R S G N Y E E Y A M D ·

701 CCTCCAGCAC AGCCTTCATG CAGCTTAACA GCCTGACATC TGAGGACTCT GCAGTCTATT ATTGTGTTAG AAGTGGTAAC TACGAAGAAT ATGCTATGGA

GGAGGTCGTG TCGGAAGTAC GTCGAATTGT CGGACTGTAG ACTCCTGAGA CGTCAGATAA TAACACAATC TTCACCATTG ATGCTTCTTA TACGATACCT

· Y W G Q G T S V T V S S T T T P A P R P P T P A P T I A S Q P L S

801 CTATTGGGGT CAAGGAACCT CAGTCACCGT CTCGTCAACC ACGACGCCAG CGCCGCGACC ACCAACACCG GCGCCCACCA TCGCGTCGCA GCCCCTGTCC

GATAACCCCA GTTCCTTGGA GTCAGTGGCA GAGCAGTTGG TGCTGCGGTC GCGGCGCTGG TGGTTGTGGC CGCGGGTGGT AGCGCAGCGT CGGGGACAGG

L R P E A C R P A A G G A V H T R G L D F A C D F W V L V V V G G V ·

901 CTGCGCCCAG AGGCGTGCCG GCCAGCGGCG GGGGGCGCAG TGCACACGAG GGGGCTGGAC TTCGCCTGTG ATTTTTGGGT GCTGGTGGTG GTTGGTGGAG

GACGCGGGTC TCCGCACGGC CGGTCGCCGC CCCCCGCGTC ACGTGTGCTC CCCCGACCTG AAGCGGACAC TAAAAACCCA CGACCACCAC CAACCACCTC

· L A C Y S L L V T V A F I I F W V R S K R S R L L H S D Y M N M T ·

1001 TCCTGGCTTG CTATAGCTTG CTAGTAACAG TGGCCTTTAT TATTTTCTGG GTGAGGAGTA AGAGGAGCAG GCTCCTGCAC AGTGACTACA TGAACATGAC

AGGACCGAAC GATATCGAAC GATCATTGTC ACCGGAAATA ATAAAAGACC CACTCCTCAT TCTCCTCGTC CGAGGACGTG TCACTGATGT ACTTGTACTG

· P R R P G P T R K H Y Q P Y A P P R D F A A Y R S R V K F S R S A

1101 TCCCCGCCGC CCCGGGCCCA CCCGCAAGCA TTACCAGCCC TATGCCCCAC CACGCGACTT CGCAGCCTAT CGCTCCAGAG TGAAGTTCAG CAGGAGCGCA

AGGGGCGGCG GGGCCCGGGT GGGCGTTCGT AATGGTCGGG ATACGGGGTG GTGCGCTGAA GCGTCGGATA GCGAGGTCTC ACTTCAAGTC GTCCTCGCGT

D A P A Y Q Q G Q N Q L Y N E L N L G R R E E Y D V L D K R R G R D ·

1201 GACGCCCCCG CGTACCAGCA GGGCCAGAAC CAGCTCTATA ACGAGCTCAA TCTAGGACGA AGAGAGGAGT ACGATGTTTT GGACAAGAGA CGTGGCCGGG

CTGCGGGGGC GCATGGTCGT CCCGGTCTTG GTCGAGATAT TGCTCGAGTT AGATCCTGCT TCTCTCCTCA TGCTACAAAA CCTGTTCTCT GCACCGGCCC

· P E M G G K P R R K N P Q E G L Y N E L Q K D K M A E A Y S E I G ·

1301 ACCCTGAGAT GGGGGGAAAG CCGAGAAGGA AGAACCCTCA GGAAGGCCTG TACAATGAAC TGCAGAAAGA TAAGATGGCG GAGGCCTACA GTGAGATTGG

TGGGACTCTA CCCCCCTTTC GGCTCTTCCT TCTTGGGAGT CCTTCCGGAC ATGTTACTTG ACGTCTTTCT ATTCTACCGC CTCCGGATGT CACTCTAACC

· M K G E R R R G K G H D G L Y Q G L S T A T K D T Y D A L H M Q A

1401 GATGAAAGGC GAGCGCCGGA GGGGCAAGGG GCACGATGGC CTTTACCAGG GTCTCAGTAC AGCCACCAAG GACACCTACG ACGCCCTTCA CATGCAGGCC

CTACTTTCCG CTCGCGGCCT CCCCGTTCCC CGTGCTACCG GAAATGGTCC CAGAGTCATG TCGGTGGTTC CTGTGGATGC TGCGGGAAGT GTACGTCCGG

L P P R *

1501 CTGCCCCCTC GCTAAGTCGA C

GACGGGGGAG CGATTCAGCT G
