# Supplementary material for: Construction and evaluation of a novel humanized HER2-specific chimeric receptor
Source: Breast Cancer Res. 2014 Jun 11;16(3):R61. doi: 10.1186/bcr3674 (PMC4095682; doi:10.1186/bcr3674)
Supplement: Additional file 2: Figure S2 — The phenotype of NT T cells cultured in vitro. (A) CD3+ T cells were the predominant cell population after 2 weeks of expansion. On day 14, PBMCs from three different donors cultured in vitro contained more that 95% CD3 + CD45+ T cells. (B) Most T cells expressed the CD8+ phenotype. (C) The NT T lymphocytes cultured in vitro were gated on CD3+ T cells and analyzed using differentiated markers CD45RO and CD62L. The phenotype of the cultured T cells from a representative donor at days 0 and 14 is shown in dot plots. [file bcr3674-S2.pptx]

## Slide 1
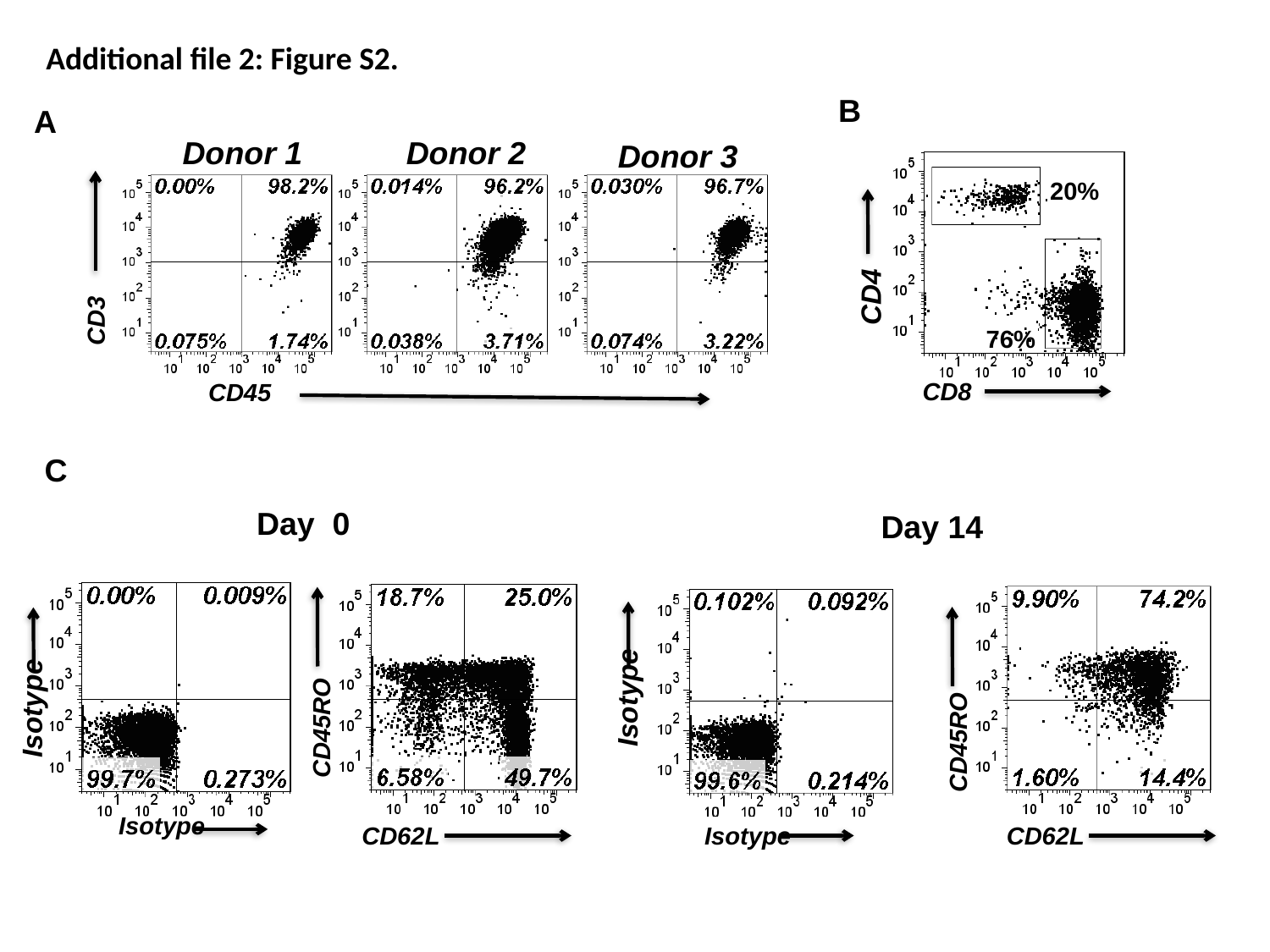

Additional file 2: Figure S2.
B
A
Donor 1
Donor 2
Donor 3
CD3
CD45
20%
CD4
76%
CD8
C
Day 0
Day 14
 Isotype
CD45RO
 Isotype
CD62L
CD45RO
 Isotype
 Isotype
CD62L
