# Supplementary figures and images for: Construction and evaluation of a novel humanized HER2-specific chimeric receptor
Source: Breast Cancer Res. 2014 Jun 11;16(3):R61. doi: 10.1186/bcr3674 (PMC4095682; doi:10.1186/bcr3674)

## Slide 1
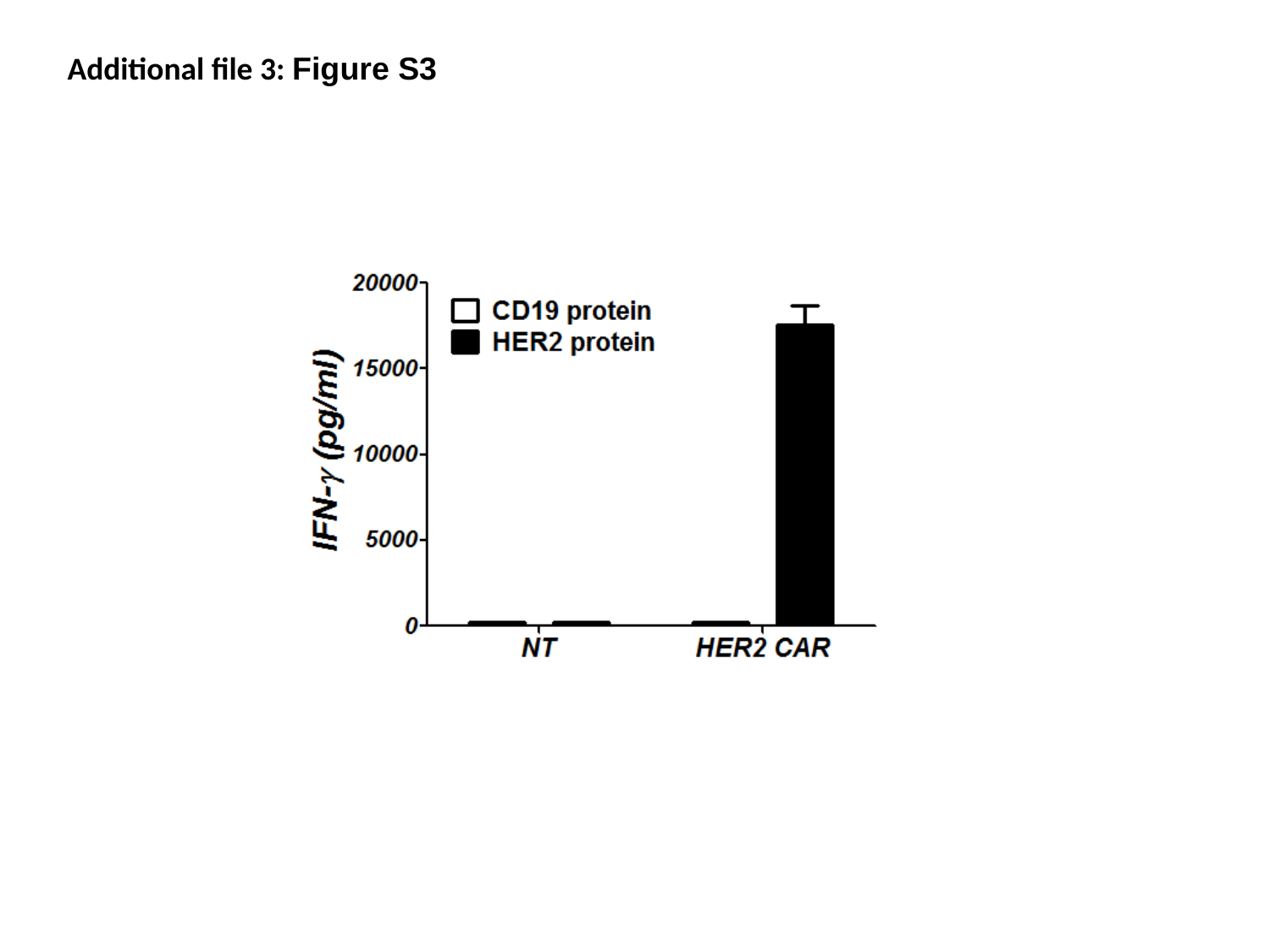

Additional file 3: Figure S3

Supplement: Additional file 3: Figure S3 — The recognition of HER2 by CAR T cells was antigen-specific. Triplicate wells of Nunc MaxiSorp MicroWell plates were coated with 5 μg/ml HER2-Fc chimeric protein or CD19-Fc chimeric protein in 200 μl of PBS overnight at 4°C. After three washings with PBS, 105 NT or CAR T cells were added, followed by incubation at 37°C. After about 24 hours, cell-free supernatants were assayed for the presence of IFN-γ. [file bcr3674-S3.pptx]
